# From representations to servomechanisms to oscillators: my journey in the study of cognition

**DOI:** 10.1007/s10071-022-01677-7

**Published:** 2022-08-27

**Authors:** Ken Cheng

**Affiliations:** School of Natural Sciences, Macquarie University, Sydney, NSW 2019 Australia

**Keywords:** Ants, Bacteria, *Paramecium*, Slime mould, Orientation, Navigation

## Abstract

The study of comparative cognition bloomed in the 1970s and 1980s with a focus on representations in the heads of animals that undergird what animals can achieve. Even in action-packed domains such as navigation and spatial cognition, a focus on representations prevailed. In the 1990s, I suggested a conception of navigation in terms of navigational servomechanisms. A servomechanism can be said to aim for a goal, with deviations from the goal-directed path registering as an error. The error drives action to reduce the error in a negative-feedback loop. This loop, with the action reducing the very signal that drove action in the first place, is key to defining a servomechanism. Even though actions are crucial components of servomechanisms, my focus was on the representational component that encodes signals and evaluates errors. Recently, I modified and amplified this view in claiming that, in navigation, servomechanisms operate by modulating the performance of oscillators, endogenous units that produce periodic action. The pattern is found from bacteria travelling micrometres to sea turtles travelling thousands of kilometres. This pattern of servomechanisms working with oscillators is found in other realms of cognition and of life. I think that oscillators provide an effective way to organise an organism’s own activities while servomechanisms provide an effective means to adjust to the organism’s environment, including that of its own body.

## Introduction

I started studying cognition, in rats, in the 1980s, in a period considered the blooming of animal cognition (Shettleworth 2010; Cheng 2016). It was a time when collected volumes on animal cognition, or comparative cognition, first appeared (Hulse et al. 1978; Roitblat et al. 1984). My focus then, and to this day, was on the mechanistic bases that allow organisms to do what they do. In recent times (Cheng 2022b; Freas and Cheng 2022), I examined, in reading literature although not in empirical research, cognitive processes in life forms beyond animals. This explains why the term “organisms” will be used frequently in this essay. The way I think about the mechanistic bases, however, has transformed over the 40-year span. For a 25th-anniversary special issue, I think it appropriate to look back over 25 years and beyond to chew over insights and major transitions in my own career. This essay documents those insights and transitions and relates them to the study of cognition at large.

I started on the goal of unpacking underlying mechanisms in the 1980s (Cheng and Gallistel 1984; Cheng 1986) by focusing on spatial cognition, as I still do today. This was a program to be differentiated—in my mind at least, if not in the words appearing in print—from what can be called success-based animal cognition, whose goal is to show what an animal can do. The success could come in a broad class of abilities, such as spatial memory (Olton and Samuelson 1976) or self-recognition (Gallup 1970). I was not alone then or now in wanting to systematically unravel how animals achieve success at the computational and behavioural levels. In my career, what that unravelling focussed on has shifted from representations to servomechanims to oscillators and actions together with servomechanistic processes. I then discovered that the last theme extends beyond animals to directed movement in non-neural organisms. The theme also extends beyond orientation and navigation to other domains of cognition and indeed of life. I aim to document this journey and the lessons that I have learned along the way. In the discussion, I consider why the theme is pervasive in life.

## Representations

**Fig. 1:**
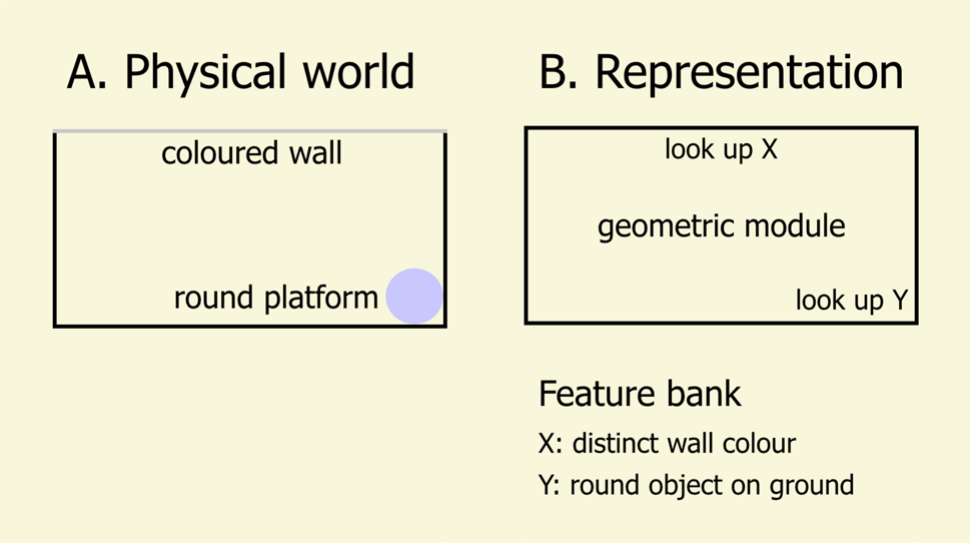
A new sketch of the nature of the geometric module proposed in Cheng (1986). **A** An overhead view of a rectangular arena with four walls, one of a different colour from the others, shown in grey. A round raised platform is in one corner. **B** The proposed representation. Only the broad geometric shape is recorded in the basic representation, the geometric module. Other notable characteristics need to be looked up via a cognitive table of features, called here the feature bank. The depiction in such illustrations does not indicate how any animal uses the representation or any action that the animal takes

When I started studying navigation, with Randy Gallistel on small-scale spatial memory in rats (Cheng and Gallistel 1984; Cheng 1986), elucidating representational content was very much the focus. The word “representation” featured in the title of both those citations: *Testing the geometric power of a spatial representation* (Cheng and Gallistel 1984) and *A purely geometric module in the rat’s spatial representation* (Cheng 1986). The theoretical focus, exemplified by the kinds of figures used to illustrate the concept of a geometric module (Fig. 1), stayed on what constructs in the head of the rat accounted for the performance of the rat. The history of the ‘geometry’ enterprise does not need to be retraced because it has been much reviewed (Cheng and Newcombe 2005; Kelly and Spetch 2012; Cheng et al. 2013; Legge 2019). Rather, what follows are a few comments in the context of the study of comparative cognition at the time.

The focus on the underlying mechanism in the Cheng (1986) study comes forth in the nature of data that likely made the paper much cited. Rats succeed in spatial tasks such as the radial maze, a maze with a central platform and narrow straight arms (typically 8) radiating from it that usually harbour food at their ends (Olton and Samuelson 1976). Olton and Samuelson’s (1976) trailblazing work established that rats used spatial memory to succeed on the radial maze, ruling out the use of smells or other intra-maze cues. Cheng (1986) attempted to further unravel the nature of the spatial memory. The study documented a peculiar error that the rat made, the rotational error. In the rotational error, diagonally opposite locations are confused. The animal might, for example, search for the round platform in Fig. 1A in the top left corner rather than the bottom right. This could arise if the animal uses only the shape in the geometric module for localisation and not the wall colours. Since then, several different theoretical perspectives have grappled with the rotational error and the ‘geometry’ literature (Cheng et al. 2013).

Cheng (1986), however, barely described the actions of the rat. What was reported were locations of digging for food, or the locations of the bottle of food that was knocked over (in the hopes that the bottle was not covered with a lid with tiny holes and sweet cereal would spill out). Neither the movements of the rat nor the digging were described. One might invoke the scenario of a young graduate student working in a cramped lab without much equipment—with no video camera, for example—as an excuse, but I believe that these limitations were not the true reason for a lack of focus on actions. Rather, the mindset was absent. Video cameras and infrared lights were available, as I used them in another unpublished study in which some male white rats roamed freely in a lab room with a specially concocted table providing puzzles for obtaining food.

## Servomechanisms

My attempts at finding mechanisms for the pigeon’s small-scale spatial cognition, much of the program in collaboration with Marcia Spetch (Cheng 1988, 1989, 1990; Cheng and Sherry 1992; Spetch et al. 1996, 1997; review: Cheng et al. 2006), did examine and describe some videotaped behaviour of the birds (Cheng 1988). This bit of focus on actions led to the idea of servomechanisms in navigation (Cheng 1995), and I still think of navigation and orientation mostly in servomechanistic terms (Cheng 2022b; Freas and Cheng 2022). The Wikipedia page on servomechanisms (https://en.wikipedia.org/wiki/Servomechanism, accessed January 2022) describes them as engineered devices designed to maintain some variable based on negative feedback. The notion has a parallel in physiology under the banner of homeostasis (Stanfield 2016), in which negative-feedback loops maintain physiological variables (e.g., cardiac output) at what we teleologically could call desired levels. In her textbook on human physiology, Stanfield (2016) called homeostasis “a primary theme throughout” (p. 31). Feedback-based regulation also figures in various areas of psychology, for example, in self-regulation (Carver and Scheier 1998) and animal learning (opponent processes: Solomon 1980; theory of reinforcement: Timberlake and Allison 1974; Hanson and Timberlake 1983). Gallistel (1980, 1981) appropriated the term to describe a basic unit of action based on negative feedback.

**Fig. 2:**
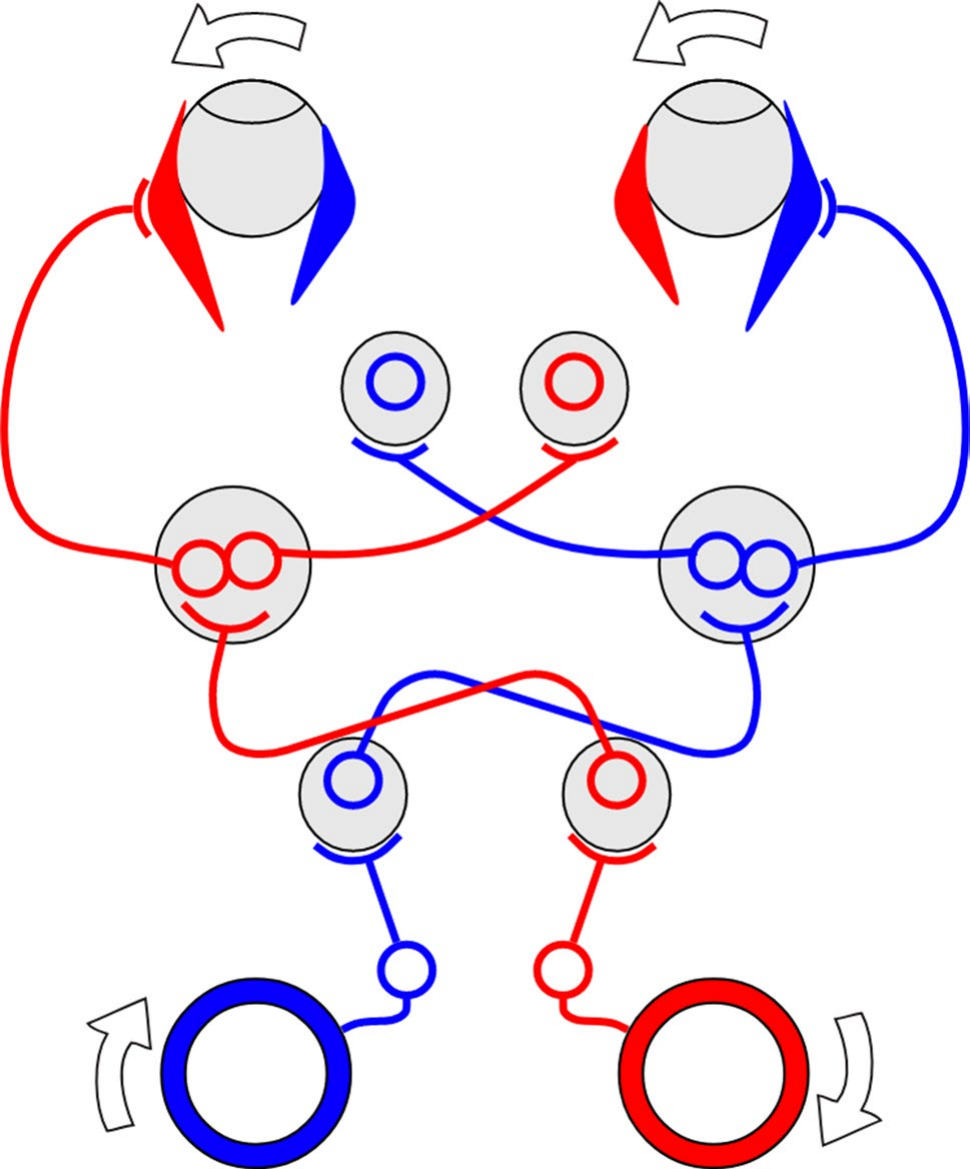
An illustration of the vestibulo-ocular reflex. The top grey circles represent the eyes of a mammal such as a human. The bottom red and blue circles represent the semicircular canals of the vestibular system in the inner ear. When the vestibular system detects a turn of the head, the eyes are counter-rotated to keep them looking at approximately the same direction in physical space. Because the eye movement does not feed back on (affect) the vestibular system, no negative feedback is delivered, making this a reflex rather than a servomechanism. Source: Wikimedia creative commons, https://commons.wikimedia.org/wiki/File:Vestibulo-ocular_reflex_blanco.svg. Author:.Koen. Licence: https://creativecommons.org/licenses/by-sa/3.0/deed.en (In colour online)

We must be careful not to equate goal-directedness in cognition and behaviour—or in physiology—with servomechanistic control systems. Servomechanisms are goal-directed, but other mechanisms may be goal-directed as well. The nature of the signal driving the action is crucial in defining a servomechanism and differentiating it from a reflex. Two kinds of visual responses in animals illustrate this distinction well (Gallistel 1981). In the vestibulo-ocular reflex, the eyes counter-rotate to rotations of the head (Fig. 2). If a human participant’s head is turned to her/his left, the participant’s eyes will turn to the right to keep looking straight ahead. This is a reflex rather than a servomechanism because the effected action, turning of the eyes, does not affect the nature of the input driving it, the vestibular signal. In contrast, the optokinetic response is a servomechanism because in this case, the effected action reduces the error that drove the response in the first place. In demonstrating the optokinetic response, the visual world is typically rotated around a test participant. For example, a vertically striped cylinder surrounding an insect might turn. The animal rotates in the direction of the rotating surround to reduce the visual slippage. In this case, the action reduces the visually based error, movement of the visual world, which triggered the action in the first place. To show that cognition is based on servomechanisms, one must demonstrate such an action–error-reduction link.

With this background on servomechanisms, it should not be difficult to think of major navigational systems as servomechanistic in nature (Cheng 1995, 2012, 2022b; Freas and Cheng 2022). Take two major strategies in an ant’s navigational toolkit, path integration and view-based navigation. In path integration, a vector representing the straight-line distance and direction from the starting point (typically, the ant’s home) is computed *enroute*. When the motivation arises to home (typically, when a morsel of food has been seized in the mandibles), the goal is to reduce the vector to zero. No matter how the vector is computed and kept online (for recent models on insects see Stone et al. 2017; Heinze et al 2018; on rodents, see Savelli and Knierim 2019), the movement reduces the error (length of the vector) that is driving the movement. In view-based navigation in ants, the goal is to recover the view at the target location (typically, home) or else to keep travelling in the direction whose view minimises some error from the best (remembered) view on the route (Cheng 2012; Zeil 2012). The ant is thought to find and keep to the best view to travel towards. Movement affects the visual input that provides the error signal to drive movement, completing the requisite loop for a servomechanism. Both path integration and view-based navigation clearly fit the conception of navigational servomechanisms.

When I first wrote about servomechanisms, however, the focus was on the representations in the central brain that support servomechanisms (Cheng 1988, 1989; theoretical position: Cheng 1995), even though servomechanisms in cognition, physiology, or even outside of the realm of life in artificial systems, must contain a component of action carried out by some effectors. In the program on small-scale spatial cognition in pigeons that Spetch and I collaborated on, the representational components were summed up in one culminating review paper (Cheng et al. 2006). The comparator system consisted of two kinds of vectors from objects and surfaces in the environment. One classic kind of vector encoded the goal as a distance and a direction from particular points in space, such as a corner of an arena. A second kind of vector encoded a perpendicular distance from a surface, such as a particular wall. Such a ‘vector’ has no defined starting point. The servomechanism driving where to peck for hidden food attempts to match multiple such vectors. The actions carried out in searching for food, aspects of the pecking motion and a few approach paths, were described a little only in one paper (Cheng 1988). Repeated pecking went through cycles identifiable as two fixations at different heights followed by a ballistic peck, consistent with earlier descriptions of pecking in pigeons (Goodale 1983). This pattern of presumably oscillatory cycles of pecking was observed to be modifiable in one bird (Cheng 1988). When in training, the food was hidden in a shallow cup that stood above ground level rather than being sunken, the bird executed more side-to-side swipes of its beak. The interpretation was that this would maximise the chances of the beak hitting the covered food cup (Cheng 1988). Oscillations were not mentioned at all. It was only recently, with a much closer focus on the actions of animals, that I, and others (e.g. Wystrach et al. 2020b; Wystrach 2021), proposed the importance of oscillatory processes in navigation, described in the next section. This last transformation can only come with a strong focus on action.

**Fig. 3:**
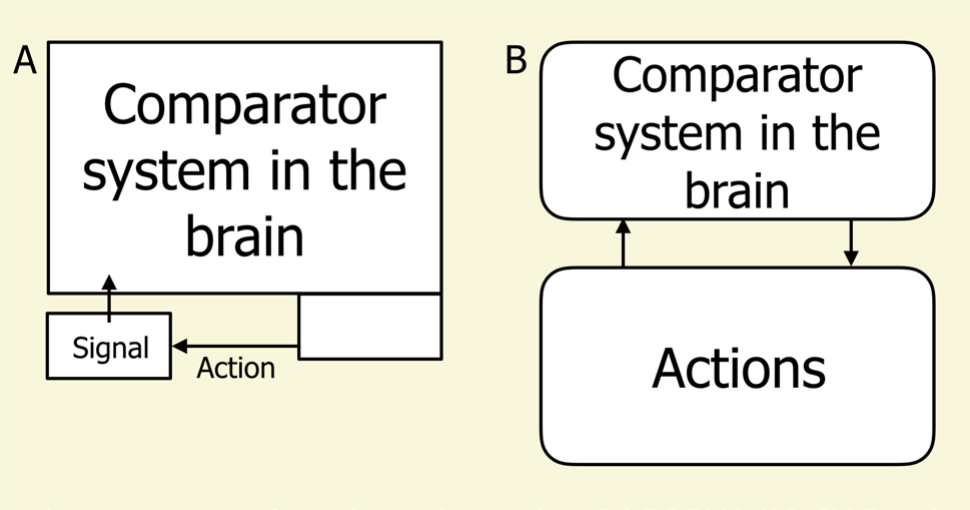
Illustrations of a servomechanism showing the proportions of space devoted to brain processes and actions from (**A**) Cheng (1995), in which the comparison process taking place in the brain took most of the space, and from (**B**) Cheng (2022b). The relative sizes of the boxes in this figure correspond to the relative sizes used in the previously published figures. The blank box in the lower right of panel A is the action generation system, which takes place in the central nervous system (brain and spinal cord)

In my study of navigation, the focus, until recently, was mostly on the nature of the representation that drives the action (Cheng 1995, 2012). In view-based navigation in ants, a palette of features is thought to be in the toolbox for use in matching, and clever experiments have documented some of this palette. By re-creating an artificial surround that mimics where the tops of terrestrial objects are found at the starting point, Graham and Cheng (2009) showed that desert ants can (and do) use what was called the skyline for view-based navigation. By manipulating views in a lab setting, Lent et al. (2013) showed evidence that wood ants use the fractional position of mass in a visual scene. This feature is the fraction of the visual scene to the left vs. to the right of the target direction. While others have paid some attention to the actions of navigating insects (e.g., Collett and Rees 1997; Lent et al. 2010), my lab had focussed on the representational side. Perhaps the best illustration of this is that in figures of servomechanisms, the comparator, the representational side, hogged the most space (Fig. 3).

A servomechanistic flavour has been given to action and cognition in history. Actions may be compared against the expected consequences of action, a notion proposed in the middle of the last century by von Holst and Mittelstaedt (1950; translated to English in Gallistel 1980, ch. 5). Actions not only do things to the world, but also generate further sensory input in what von Holst and Mittelstaedt called reafference. In executing actions, the brain needs to take account of reafference. One notion that von Holst and Mittelstaedt proposed for this accounting is an efference copy. This is a signal to other parts of the brain that certain motor commands have been issued. To use an organisational analogy, an efference copy is akin to an email to the boss saying that we have told the team to do X; expect changes by way of X′. Actions may be compared against expected consequences. Other notions with a servomechanistic flavour followed.

A decade after von Holst and Mittelstaedst’s seminal (1950) idea of reafference and two decades before Gallistel’s (1980) formulation of basic units of action, cognition was cast in servomechanistic terms in the form of a test-operate-test-exit unit (Miller et al. 1960), considered a basic unit of action and cognition. The system *tests* for conditions for the execution of a particular action (*operate*). It keeps testing until the conditions no longer call for action, at which point an *exit* is made.

In the control of action, Powers (1973, 1978) also championed a servomechanistic view, with servomechanisms called “control systems”. Servomechanisms are nested hierarchically into levels, with higher-level systems exerting control over lower-level systems by adjusting their set points (Powers 1973). The title of Powers’ (1978) article suggested that the author considered this notion basic: in the subtitle stood the phrase “Foundations of Scientific Psychology” (p. 417). Powers (1978) criticised others for dismissing the notion of control systems—servomechanisms—for being machine-like and, thus, simplistic. Rather, he claims that it is the other way around: Engineers borrowed the idea of control systems from the study of life and built for our convenience necessarily simple systems, compared with any form of life. All the empirical data that Powers presented consisted of humans controlling what appears on the screen of a monitor. Thus, one can question whether such a base forms a foundation for scientific psychology. But my view is that the notion of a servomechanism will form part of the foundation for all life sciences. I explain further in the Discussion.

**Fig. 4:**
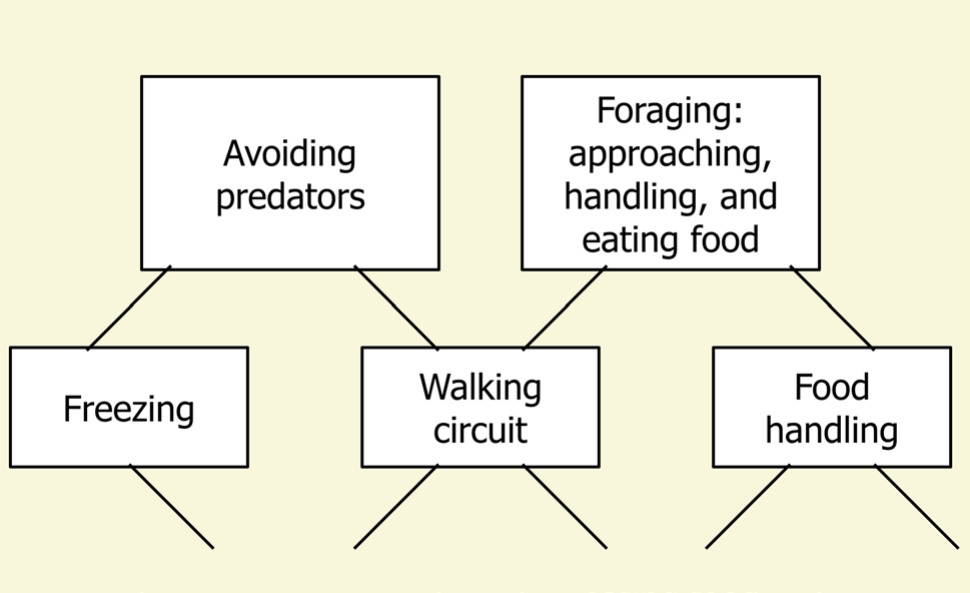
A simple illustration of a lattice hierarchy. The key ingredient defining a lattice hierarchy is that different higher-level units may command the same lower-level unit. In the figure, motivational systems of foraging and predator avoidance both call on circuits orchestrating walking

Gallistel (1980) also proposed the notion of hierarchical control of action under the term lattice hierarchy (Fig. 4). A lattice hierarchy is not a strict hierarchy. In a lattice hierarchy, a lower-level unit may be under the command of multiple higher-level units, with different higher-level units calling on the same lower-level circuits to achieve goals. In the simple example in Fig. 4, the walking circuit is used by two higher motivational systems, to move away from danger or to approach food, while other circuits are peculiar to one of those systems. Handling is an action done on food, not on dangerous items such as predators.

More recently, Buzsáki (2019) pushed for a view of neuroscience that takes seriously the nervous system’s own properties and own ways of doing things, rather than merely treating it as a device reacting to input stimuli. One could call this a more intrinsic view of the nervous system, although Buzsáki’s (2019) title is more evocative: “The Brain from Inside Out”. Many of the intrinsic properties of the brain come from the endogenous oscillations in the brain, the various brain waves given Greek letters, such as alpha, beta, and theta, as names. I will save discussion on these oscillatory waves for later, turning now instead to a notion of servomechanism that I have drawn from Buzsáki’s (2019) book.

Buzsáki (2019) characterised cognition–action links in what I take to be servomechanistic terms. Thus, he writes of overt actions (p. 104, Fig. 5.1) that the.

“output moves sensors, which scan the surroundings or the body so that the brain can predict the consequences of its action based on prior experience (phylogenetic or ontogenetic) in similar situations.”

I take this to mean that a comparison process akin to what a comparator in a servomechanism does is inherent in the actions of a range of animals with brains. Later, Buzsáki (2019) thinks of internalised actions, activities in the brain, in a similar way:

“The key physiological mechanism … is a corollary discharge-like system that allows the brain to interpret the activity of its action circuits even in the absence of overt movement and sensory feedback from muscles.” (p. 138)

A corollary discharge is another term for an efference copy. This passage implies that such a comparison process takes place with covert actions as well, a notion proposed by von Holst and Mittelstaedt for overt action (1950; translated in Gallistel 1980, ch. 5). Buzsáki’s more recent (2019) ideas on action perhaps echo von Holst and Mittelstaedt.

## Oscillators

Oscillators are considered another basic unit of action along with the reflex and the servomechanism (Gallistel 1980, 1981). All these units generate action and are identifiable across many realms of behaviour, but they operate in different ways. A reflex generates some stereotypical action to a restricted class of stimuli, adequate stimuli. The servomechanism, as we have seen, generates action to reduce some error. The oscillator contains two key properties: it generates action endogenously and it produces periodic action. In neurally endowed organisms, a pacemaker is crucial to orchestrating an oscillator. A pacemaker is a component of a neural circuit—and it can be a single neuron—that fires periodically and regularly without the need for input. The pacemaker generates periodic actions via effectors. In non-neural organisms, the definition of an oscillator must be broadened to any pattern of periodic action, however they are generated. These generative mechanisms are varied and not all well understood (Cheng 2022b).

**Fig. 5:**
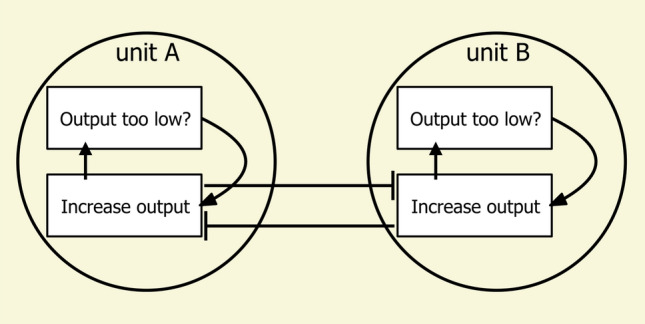
A conceptual scheme for mutually inhibitory oscillators, adapted from Clement et al.’s (2022) preprint (Fig. 8A). Two units, which may be conceived as single neurons, each attempt to regulate its output at a certain level. The two units, however, inhibit one another’s output, indicated by the sideways-T arrows. The end result is that the output of each unit waxes and wanes over time in seesawing antiphase to one another (A up, B down; B up, A down)

Oscillators may be fashioned out of servomechanisms. As an everyday example, think of the operation of a typical thermostat in heating a room. Does the thermostat generate a steady and continuous stream of heat? No, it triggers heating periodically, when the room temperature dips too low. It then stops once the temperature creeps too high. The end result is periodic cycles of heating producing regular, hopefully small oscillations in room temperature around the set point. A recent conceptual example comes from a proposal for how navigating ants generate transverse (left–right) oscillations in walking (Fig. 5; Clement et al. 2022 preprint). Two proposed endogenous units govern left and right turns by regulating themselves in servomechanistic fashion to output at a steady rate. The two units, however, mutually inhibit one another. We end up with a cycle: when A reaches its output set point, it decreases B’s output below its set point, which causes B to increase its output, which inhibits A and brings A’s output below its set point, which causes A to increase its output, which inhibits B … Empirically, several genera of ants that have been examined in enough detail do oscillate in navigation (*Myrmecia*: Murray et al. 2020; Clement et al. 2022 preprint; *Iridomyrmex*: Clement et al. 2022 preprint; *Melophorus*: Wystrach et al. 2011, in which it was called “wiggling” and “meandering”). The Meander in Wystrach et al. (2011) was a formal measure of how much each segment covering 30 cm of straight-line distance changed direction from the direction of the previous 30-cm segment; “wiggling” was an informal term. This topic is a current frontier in ant navigational research, and I expect more research on the nature of oscillations in ant navigation in the years to come.

Other organisms rely on oscillators to move as well, from giant sea turtles to single-celled organisms (Cheng 2022b). Sea turtles swim by rotating (oscillating) the front flippers in a synchronous power stroke resembling a butterfly swimming style (Salmon and Wyneken 1987). In the multi-nucleated single-celled slime mould *Physarum polycephalum,* muscle-like fibres composed of actin and myosin contract the cell walls to shuttle internal fluids about in an orderly periodic manner (Avsievich et al. 2017; Boussard et al. 2021). Vein-like tubules inside the body also contract and shunt fluid about (Kramar and Alim 2021). In eukaryotic single cells such as *Paramecium*, cilia on the outside beat like a team of coordinated oars to propel the organism. The cilia do not beat each to its own rhythm, but coordinate themselves in a Mexican-wave-styled temporal order called meta-chronal waves. One cilium starts to beat. The next cilium then begins its beating, closely followed by its neighbour, etc. The whole team creates a wave of beating from front to back or from back to front with respect to the direction of travel. Half a century ago, discussion concerned whether external factors such as hydrodynamic flow caused by the beating of one cilium or internal, physiological factors sometimes termed “neuroid” processes drive the coordinated beating. The suspicion was that both kinds of factors are at play (Kinosita and Murakami 1967; Sleigh 1969). With more sophisticated modelling half a century later, these two kinds of factors are still discussed (Wan and Goldstein 2016; Hamilton et al. 2019), with perhaps different organisms relying more on one kind vs. the other (Hamilton et al. 2019). In the bacterium *Escherichia coli*, the constant turning of a motor bundles up the flagella together into a sperm-like tail to beat in coordination to propel the prokaryote forwards (Koshland 1980). The bundling and locomotion result when the motor turns counterclockwise.

A key point about these locomotory oscillations is that they do not just beat willy-nilly of their own accord, but are sensitive to various feedback mechanisms. In short, servomechanisms could manipulate the oscillations. I only came upon this notion in 2020 (Cheng 2022b; Freas and Cheng 2022) and have yet to explore the full extent of this theme. What follows are some highlights. Orientation and navigation in organisms can be characterised as servomechanisms working with oscillators. So can other realms of cognition, and indeed other realms of life.

## Servomechanisms working with oscillators

### Orientation and navigation

The theme of servomechanisms working with oscillators applies across all scales of earthly travel, from microscopic bacteria in the range of micrometres to ocean-spanning sea turtles in the range of thousands of kilometres. The latter world travellers adjust the cyclic flapping of their flippers in response to experimentally created rotational disturbance (Avens et al. 2003; Cheng 2022b). With a roll (turn around the front-to-back axis), the two front flippers stroke at different depths beneath the turtle’s body. In my mind, this theme first emerged in my main area of recent research, ant navigation, more than a decade ago (Wystrach et al. 2011), but signs of undulating paths can be seen in earlier work (e.g. Graham et al. 2003; Durier et al. 2004). Ants do not always walk straight in some direction, but “meander”, as it was (and sometimes still is) called (Wystrach et al. 2011). This was a behaviour reflecting what we thought was uncertainty, likely not a direct measure of uncertainty in the desert ant’s brain, but some proxy stemming in some way from uncertainty in the experimentally manipulated situations. In Wystrach et al.’s (2011) study, positions of experimentally provided landmarks and of the travelling ant were both manipulated. We did not, however, look systematically at the nature of the meandering.

**Fig. 6:**
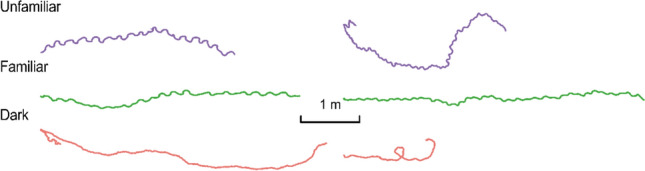
Examples of left–right or transverse oscillations in diurnal bull ants *Myrmecia croslandi* (left) and meat ants *Iridomyrmex purpureus* (right) walking on a trackball placed at different locations. The trackball consists of a styrofoam sphere floating on air. Ants support their own weight on the trackball while walking. The presence of lateral or transverse oscillations is strongest in the unfamiliar environment and weakest in the dark. From Clement et al. (2022). Open-access licence: https://creativecommons.org/licenses/by-nc-nd/4.0/. (In colour online)

In this decade, the oscillatory nature of ant locomotion became clear (Murray et al. 2020; Le Moel and Wystrach 2020; Wystrach et al. 2020b). A closer examination by eye—beyond merely tabulating a meander measure—reveals the bigger and more obvious transverse oscillations. However, putting ants (bull ants and meat ants) on a floating styrofoam ball and videotaping their walking reveal finer oscillations (Murray et al. 2020; Clement et al. 2022 preprint; Fig. 6). These transverse oscillations are superimposed on another set of coupled oscillations that coordinate leg movements to propel the ant forwards (Wilson 1966; Gallistel 1980). The most common gait is the tripod gait, in which the middle leg on one side (e.g. left) steps in approximate synchrony with the front and rear legs on the opposite side (e.g. right; Pfeffer et al. 2019; Tross et al. 2021).

Both these sets of oscillations are modified in light of circumstances, that is, they come under servomechanistic control (Wystrach et al. 2019, 2020a; Clement et al. 2022 preprint). One rule at play is: in more uncertain circumstances, however that is determined by the ant, slow down and meander more. Thus, the frequency of the tripod gait decreases while the amplitude of the transverse oscillations increases. One way to increase meander is to capture a homing desert ant and place it back at some point on its homing route, a procedure called rewinding (Wystrach et al. 2019). Another way to do so is to present a pit trap filled with debris that delays a homebound trip (Wystrach et al. 2020a). Desert ants subsequently meander more before they reach the trap area.

At this point, the ways that both these sets of oscillations (left–right weaving and tripod gait) change with circumstances have yet to be well characterised, and I urge a focus on action as a program of research in navigation, and indeed in other domains of comparative cognition. Such a notion as servomechanisms operating on oscillations only came about from examining action in some detail, not simply focusing on what the organism can achieve.

Tiny single-celled organisms, both prokaryote and eukaryote, also orient by this theme (Cheng 2022b; Freas and Cheng 2022), but with a difference in the mode of operation. The link between the servomechanism and the oscillator is less intricate and more incidental. Forward motion is occasionally interrupted in servomechanistic fashion, with oscillators happening to be the agents propelling forward movement. These tiny denizens orient rather than navigate. In orientation, an organism moves—typically up or down some gradient that it can sense—to get to a better place. The organism is not aiming for any particular place; any better place will do. This is the sense of orientation that Fraenkel and Gunn described in their (1961) book on animal orientation. In navigation, on the other hand, an organism attempts to reach one particular place, for example, the one nest that is an ant’s own nest. A common theme is chemokinesis effected by occasionally interrupting forward movement propelled by oscillations. As discussed already, bacteria move by a constantly turning motor that bundles the flagella together into an oscillating tail (Koshland 1980). The flagella bundle up when the motor turns counterclockwise. Occasionally, the motor stops and then turns in the opposite, clockwise, direction. The flagella then come apart and the *E. coli* bacterium takes a spin called a tumble (Koshland 1980) or twiddle (Berg and Brown 1972) that orients it in a random direction. The rate of tumbles is adjusted to orient up a chemical gradient (Koshland 1980; Cheng 2022b). When the gradient improves with travel, the rate of tumbles decreases; when things do not improve or get worse, the rate of tumbles increases. *Salmonella enterica* has a more efficient way to control the tumbles that improves its efficacy: when the going gets good, the tumbles are biased to be small—thought to be caused by unravelling less than all the flagella—resulting in more small turn angles than expected by chance (Nakai et al. 2021). The eukaryote *Paramecium* also engages occasionally in its version of tumbles, called avoiding reactions (van Houten 1978) because that it what it does after bumping into something. (In proper learning terminology, this is escape rather than avoidance, but the term “avoiding reaction” has stuck.) *Paramecium* springs back, with its cilia rowing in the opposite direction to usual, and then reorients in a random direction. The rate of avoiding reactions is, like in the prokaryotes, adjusted in chemokinesis.

The mechanism is often called, too loosely to my mind, chemotaxis, but it is properly termed “chemokinesis”. In a taxis, the servomechanism adjusts the direction of travel based on available information to orient in a better (if not optimal) direction of travel. In a kinesis, in contrast, only the rate of something, such as tumbles, is adjusted, with no guarantee that the new direction is a better direction of travel. Nevertheless, kinetic mechanisms still function to guide the organism to a locally optimal location, for example, the best concentration of food in the locale.

The neurally endowed nematode *Caenorhabditis elegans* (with 302 neurons) also engages in its version of tumbles, called turns or pirouettes (Pierce-Shimomura et al. 1999; Srivastava et al. 2009; Sterling and Laughlin 2015; Tanimoto and Kimura 2019). The rate of turns is similarly adjusted in light of the chemical gradient in chemokinesis. The small worm possesses other mechanisms to boost its performance, including true chemotaxis in a mechanism called weathervaning (Iino and Yoshida 2009; Tanimoto and Kimura 2019). The worm’s head swings left and right, and the side with the better chemical gradient attracts bigger turns in that direction (thus: left side better gradient, turn left more than turn right).

I will leave this subsection with these highlights (for reviews, see Cheng 2022b; Freas and Cheng 2022) and end with a brief discussion. With the cliché about the acuteness of hindsight echoing in the background, it is not surprising that orientation and navigation rely on modifying oscillators. Oscillators typically drive locomotion, from flagella to cilia to the limbs of animals. It makes sense for orientational and navigational mechanisms to work on these drivers. The servomechanisms, however, differ in sophistication, with transverse oscillations (moving side to side) endowing the traveller with significant improvements. *E. coli*’s flagellar beating propels it straight ahead, and it can only adjust its locomotion by varying the rate of interruptions, resulting in chemokinesis. A *Drosophila* larva oscillates transversely (Wystrach et al. 2016) and a *C. elegans* worm swings its head transversely (Iino and Yoshida 2009; Tanimoto and Kimura 2019). All these organisms rely on a short-term memory of the chemical gradient, but the transversely oscillating organisms can adjust their direction of locomotion to a better direction based on their memory, resulting in chemotaxis rather than chemokinesis. Left–right oscillations are now thought to give ants a mechanism for ensuring that they are keeping to an optimal course of travel (Le Moel and Wystrach 2020;; Wystrach et al. 2020b). One intuitive line of argument runs as follows: if you keep travelling in one direction, how can you be sure that that is the best direction of travel, as you have no other direction to compare with? Transverse oscillations allow the ant to keep adjusting its course back to the optimal direction. Much more theoretical and empirical work is needed on this new front.

### Cognition in mammals

Especially in the past two decades, oscillatory processes have been reported in diverse realms of cognition in mammals (Crystal 2006; Lisman and Jensen 2013; VanRullen 2016, 2018; Buzsáki 2019; Pomper and Ansorge 2021), including timing, perception, attention, and short-term memory. Many of these oscillatory processes would fit into the theme of servomechanisms working with oscillators. The theme includes working with both motor systems and with covert operations within the brain. A few highlights follow.

Starting with motor control, when a rat has to navigate to places on the radial maze (Olton and Samuelson 1976) or in a digging task (Cheng 1986), it needs to move its limbs. These limbs move in an orderly oscillatory fashion (Thota et al. 2005). In walking, 3D kinematics revealed orderly cycles in each limb, with the four limbs also moving in a cyclic order: left rear limb lifts, then left front limb, then right rear limb, then right front limb. The two pairs of diagonally opposite limbs lift off closer in time than do the two limbs on one side (Thota et al. 2005, their Fig. 2). The sequence parallels the slowest gait in insects (Wilson 1966; Gallistel 1980), in which these arthropods also lift one limb at a time, first in sequence on one side, then in sequence on the other side. To cope with environmental slope, the rodent gait is adjusted (Li et al. 2021). Navigational servomechanims must also work with the oscillatory cycles of limb movements to reach destinations, a theme well worth researching in not just rats, but in other vertebrate animals too.

One pattern of data from some tasks of cognition consists of periodic waxing and waning in performance on a short timescale with typically sub-second periods. Similar cycle frequencies are found in visual and other modalities across many studies, with 10 Hz and 7 Hz standing out as notable peaks (VanRullen 2016). Hints of periodicity in cognition were reported as early as 1960 in reaction time (Venables 1960), but it takes not only sophisticated data gathering, but also sophisticated data analysis to reveal periodicities clearly and convincingly (VanRullen 2016). Some examples best illustrate.

In one task requiring short-term memory, students had to keep in mind two different grid orientations of light and dark sine-wave patterns that have featured in many studies of cognition (Pomper and Ansorge 2021). If either one of those grid orientations was shown as a stimulus, they had to indicate its presence by pressing a button. Cycles of performance on the two stimulus orientations, waxing and waning in level, were found. In neurophysiology, a number of oscillations within the brain that bears on cognitive performance have been proposed or identified.

One proposal for organising navigation states that different portions of the theta cycle in rodent brains provide different kinds of navigational information (Sanders et al. 2015, 2019). Place cells firing in the first half of each cycle encodes where the rodent is currently, while in the second half of the cycle, the firing of place cells project ahead to where the rodent is headed to. As a rodent moves through a place field, a place cell fires earlier and earlier in the theta cycle, a phenomenon known as (theta) phase precession.

In a detection task requiring attention in rhesus monkeys, the frontal eye fields and the lateral parietal area play crucial roles in driving cycles of performance (Fiebelkorn et al. 2018). The primates had to pay covert attention to a signalled spot on a screen at which a stimulus will appear with 78% probability. Eye movements were not allowed in doing the task. The contrast of the stimuli to be detected was adjusted to be near detection threshold. Simultaneous neurophysiological recording from the cortex allowed the authors to deduce which brain regions and which rhythms drove the cycles of better and poorer performance found in the behaviour of the macaques. The performance cycle followed the theta rhythm, 3–8 Hz. Two different sources drove the better and poorer parts of the performance cycle. The theta rhythm in the frontal eye fields was a key driver of the ‘good’ phase while the alpha rhythm in the lateral intraparietal region led the ‘poor’ phase. Such performance cycles are also found in humans, both in detecting near-threshold stimuli and in reaction time to supra-threshold stimuli (Helfrich et al. 2018).

In sum, a large swath of cognition shows cycles suggesting that underlying oscillators are at work. I would suggest the broad hypothesis that much of mammalian cognition consists in servomechanistic systems adjusting oscillatory systems for task-driven purposes. It would be interesting for the field of comparative cognition to look routinely for cycles in performance as well as at details of movements in animals doing experimental tasks.

### Cognition in slime mould

**Fig. 7:**
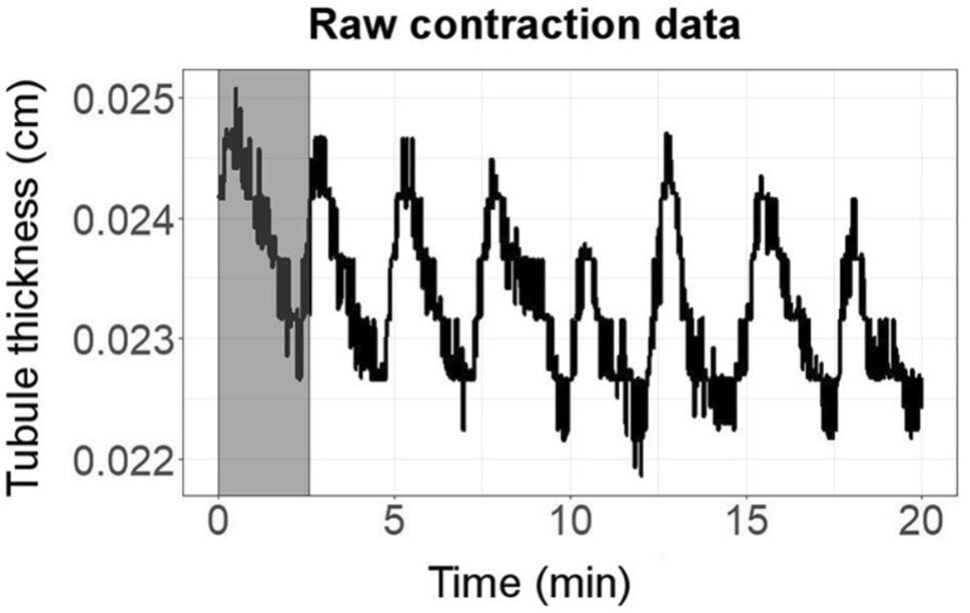
An example of oscillatory cycles in *Physarum polycephalum*, in the thickness in a tubule at a location of experimental interest. Note the time scale of minutes on the *x*-axis. From Ray et al. (2019). Open access: distributed under the terms of the Creative Commons Attribution License (CC BY)

The slime mould *Physarum polycephalum* has been called “the intelligent unicellular eukaryote” (Kramar and Alim 2021, p. 1) because it can accomplish many cognitively challenging feats. It solves mazes, finds high-quality solutions to the travelling-salesperson problem, forms optimised networks likened to the Tokyo subway system, and uses its body as a memory device (de la Fuente 2015; Reid et al. 2015; Reid and Latty 2016; Smith-Ferguson and Beekman 2020). The slime mould in its plasmodium stage is multi-nucleated and moves by oozing protoplasm in pseudopods that can extend in any direction. Its core business is based on oscillations of protoplasm controlled by structures made of actomyosin—thus using the actin and myosin that make up vertebrate muscles—in the cell wall and in tubules within its body. These muscle-like structures contract and relax to make oscillations of the protoplasm inside (Fig. 7). Modifications of these oscillations allow *Physarum* to do its problem solving in time, often clocked in hours rather than seconds.

A full account of all these processes would require too much space, but examples can be given. A bodily memory can be created by increasing the tubule diameters in the area that contacts food (Kramar and Alim 2021; comment: Cheng 2022a). The part of the body that touches food ends up with tubules of larger diameters, hence with more protoplasm, as a function of some hypothesised loosening agent. The amount of protoplasm in the body being constant, other areas contract. Modifications of the pattern of oscillations make this a bodily form of memory. When offered protein and carbohydrates separately, the slime mould adjusts its biomass to take in an optimal ratio of the two macronutrients (Dussutour et al. 2010). More recently, Dussutour and colleagues proposed that oscillations are key to learning and adaptive behaviour in *Physarum* (Boussard et al. 2021). The theme of adjusting oscillations to pursue various ends seem to fit the life of *Physarum polycephalum*, well beyond orientation and navigation.

## Discussion

Two questions regarding this brief tour of servomechanisms working with oscillators come to mind. Why is this mode of operation so pervasive in life and why has this not been obvious much earlier? This discussion centres around these points.

To start with the second question in the realm of cognition, I think that a focus on achievement and representation has dampened progress because we have not studied enough of the details of what organisms are doing. In part, this is because achievement measures are easy to tabulate. Did the animal solve the task on this trial? How many different arms on the radial maze did the animal visit in its first eight choices? Was the initial heading direction of the ant close to the goal direction? As a good deal of my group’s research had focussed on this last question (e.g. Graham and Cheng 2009; Schultheiss et al. 2016), I am by no means excepting myself. Radial-maze performance in rats and initial headings in ants make good illustrations of the point that I am making, which is that interesting and important ideas could emerge when the actions of animals are observed in detail.

On the radial maze, much research in rats focussed on how well the animals performed and on ruling out alternative explanations to the use of spatial memory (Olton and Samuelson 1976; Roberts 1984). Brown (1992) examined and reported on how rats made their choices of arms on the radial maze. The rats looked down each arm, called a micro-choice, before deciding to go down the arm, called a macro-choice, or go to another arm. Macro-choices, typically used to measure performance and typically all that are reported, were well above chance levels at being unvisited arms. Micro-choices, on the other hand, were close to random choices. Brown suggested that although rats are likely to form cognitive maps of their environment, the status of whether an arm had been visited might not be on these maps. At least the data are consistent with this view.

Ants use surrounding landmarks for homing and are thought to use a palette of visual features. Experiments that only measured initial headings (e.g. Graham and Cheng 2009) did not note the oscillatory nature of ant locomotion. It was from examining in some detail the paths that the ants took (Le Moel and Wystrach 2020; Murray et al. 2020) that this oscillatory nature became clear. The representational basis of navigation and of cognition is an important component of the story that I do not wish to downplay. In fact, I still think that we cannot dispense with the notion. But representations form only a part of the story. It is high time we looked closely at the actions that organisms are taking.

As for the reasons why servomechanisms working with oscillators form a pervasive pattern in life, I conjecture that servomechanisms and oscillators make effective means of conducting two kinds of business essential to life, that of organising the organism’s own activities and responding effectively to the environment, including the organism’s own internal environment. Oscillators are used to organise many activities while servomechanisms help an organism to adapt to its environment in ways that maintain life. I expand on these points briefly but also set the conjecture forth as a broad program of empirical and theoretical (or literature-based) research.

In locomotion, the need to coordinate effectors carrying out the deed is perhaps easiest to appreciate. In the required coordination, flagella, cilia, limbs, and wings all need two kinds of regularities: they need to move in a regular cyclic manner, and the beats of separate effectors need to be timed appropriately, meaning some coordination between the cycles of different locomotory effectors. Insect gait (Wilson 1966; Gallistel 1980) and the coordination of cilia in eukaryotes (Wan and Goldstein 2016; Hamilton et al. 2019) have already been discussed above.

In the multicellular nervous systems of mammals, coupling of different oscillators is thought to help represent multiple items in an orderly way (Lisman and Jensen 2013) or gate attention (Fries 2015). Another possible functional advantage is saving energy using neuronal resources periodically (VanRullen 2016). It is also possible that the operation of a nervous system may be impossible without periodicities (VanRullen 2016). Processes of life being complex even in what we may call “simple” organisms—mistakenly in my mind—such as bacteria, this last hypothesis of VanRullen’s presents a broad theoretical framework to explore in other systems, including non-neural life.

Another business that all life must conduct is to adjust to environmental circumstances, including the environment within an organism’s body. Servomechanisms are pervasive in adjusting to environmental conditions, forming a key basis for regulation. A negative-feedback loop does not simply set off a process, but tracks how the process is going and shuts off when the error signal driving it has waned. Regulation is preferable to only setting off a reaction with no mechanism for reining that reaction in again. Life cannot operate as the Sorcerer’s Apprentice did. In the famous cartoon in the movie *Fantasia* (Algar 1940; music by Paul Dukas, starring the world’s most famous cartoon rodent), the Sorcerer’s apprentice had the task of fetching buckets of water. The clever but unwise apprentice set a broom off fetching water but had no idea how to stop the broom, leading to disastrous flooding consequences. Life cannot act like the Sorcerer’s apprentice; no Sorcerer will appear in the nick of time to fix runaway catastrophes. In general, life processes must be regulated.

When it is functioning properly, the mammalian brain does not operate like the Sorcerer’s apprentice. Circuits that rely on various neurotransmitters often contain auto-receptors that are sensitive to the neurotransmitter used by that particular circuit. Auto-receptors feed back on the circuit and are inhibitory in nature; they have been found for serotonin (Roberts et al. 2001; Albert and Vahid-Ansari 2019), somatostatin sst_1_ (Thermos et al. 2006), and dopamine (Nolan et al. 2020). It might seem unintuitive or even perverse to put brakes on a circuit as soon as it is excited, but the metaphor of the Sorcerer’s Apprentice provides an explanation for why the brain, to put it loosely, ‘worries’ about stopping processes as well as starting processes.

## Conclusion

I have set forth servomechanisms and oscillators as fundamental processes in orientation and navigation. Oscillators propel locomotion in organisms, while servomechanisms work with oscillators to achieve goals in orientation and navigation. This theme applies to more than orientation and navigation. It applies to cognition, in unicellular organisms such as the slime mould *Physarum polycephalum* (Reid and Latty 2016; Boussard et al. 2021) as well as mammals, in which rhythms of the brain are crucial to cognition (Buzsáki 2019). The full range of the interplay between servomechanisms and oscillators has yet to be explored. We should not become too giddy, however, and claim that everything in life runs on oscillators and servomechanisms. Life is likely far more complex than any one of us imagines. But oscillators provide good ways for an organism to organise its own activities while servomechanisms help an organism to adjust to its environment, including the environment inside its own body. Together, these two processes make key players on team Life.

## Data Availability

This paper does not contain any original data.

## References

[CR1] Albert PR, Vahid-Ansari F (2019). The 5-HT1A receptor: signaling to behavior. Biochimie.

[CR2] Algar J (1940). The Sorcerer’s apprentice.

[CR3] Avens L, Wang JH, Johnsen S, Dukes P, Lohmann KJ (2003). Responses of hatchling sea turtles to rotational displacements. J Exp Mar Biol Ecol.

[CR4] Avsievich TI, Frolov SV, Proskurin SG (2017). Interrelation between respiratory and contractile activity of *Physarum polycephalum*. J Phys D Appl Phys.

[CR5] Berg HC, Brown DA (1972). Chemotaxis in *Escherichia coli* analysed by three-dimensional tracking. Nature.

[CR6] Boussard A, Fessel A, Oettmeier C, Briard L, Döbereiner H-G, Dussutour A (2021). Adaptive behaviour and learning in slime moulds: the role of oscillations. Philos Trans R Soc B.

[CR7] Brown MF (1992). Does a cognitive map guide choices in the radial-arm maze?. J Exp Psychol Anim Behav Process.

[CR8] Buzsáki G (2019). The brain from inside out.

[CR9] Carver CS, Scheier MF (1998). On the self-regulation of behavior.

[CR10] Cheng K (1986). A purely geometric module in the rat’s spatial representation. Cognition.

[CR11] Cheng K (1988). Some psychophysics of the pigeon’s use of landmarks. J Comp Physiol A.

[CR12] Cheng K (1989). The vector sum model of pigeon landmark use. J Exp Psychol Anim Behav Process.

[CR13] Cheng K (1990). More psychophysics of the pigeon’s use of landmarks. J Comp Physiol A.

[CR14] Cheng K, Medin DL (1995). Landmark-based spatial memory in the pigeon. The psychology of learning and motivation.

[CR15] Cheng K, Zentall TR, Wasserman EA (2012). Arthropod navigation: ants, bees, crabs, spiders finding their way. The Oxford handbook of comparative cognition.

[CR16] Cheng K (2016). How animals think and feel: an introduction to non-human psychology.

[CR17] Cheng K (2022). Bodily memory in slime mold. Learn Behav.

[CR18] Cheng K (2022). Oscillators and servomechanisms in orientation and navigation and sometimes in cognition. Proc R Soc B-Biol Sci.

[CR19] Cheng K, Gallistel CR, Roitblat HL, Bever TG, Terrace HS (1984). Testing the geometric power of an animal's spatial representation. Animal cognition.

[CR20] Cheng K, Newcombe NS (2005). Is there a geometric module for spatial orientation? Squaring theory and evidence. Psychon Bull Rev.

[CR21] Cheng K, Sherry DF (1992). Landmark-based spatial memory in birds (*Parus atricapillus* and *Columba livia*): the use of edges and distances to represent spatial positions. J Comp Psychol.

[CR22] Cheng K, Spetch ML, Kelly DM, Bingman VP (2006). Small-scale spatial cognition in pigeons. Behav Proc.

[CR23] Cheng K, Huttenlocher J, Newcombe NS (2013). 25 years of research on the use of geometry in spatial reorientation: a current theoretical perspective. Psychon Bull Rev.

[CR24] Clement L, Schwarz S, Wystrach A (2022). An intrinsic oscillator underlies visual navigation in ants. bioRxiv.

[CR25] Collett TS, Rees JA (1997). View-based navigation in hymenoptera: mulitple strategies of landmark guidance in the approach to a feeder. J Comp Physiol A.

[CR26] Crystal JD (2006). Time, place, and content. Compar Cogn Behav Rev.

[CR27] De la Fuente IM (2015). Elements of the cellular metabolic structure. Front Mol Biosci.

[CR28] Durier V, Graham P, Collett T (2004). Switching destinations: memory change in wood ants. J Exp Biol.

[CR29] Dussutour A, Latty T, Beekman M, Simpson SJ (2010). Amoeboid organism solves complex nutritional challenges. Proc Natl Acad Sci USA.

[CR30] Fiebelkorn IC, Pinsk MA, Kastner S (2018). A dynamic interplay within the frontoparietal network underlies rhythmic spatial attention. Neuron.

[CR31] Fraenkel GS, Gunn DL (1961). The orientation of animals.

[CR32] Freas CA, Cheng K (2022). The basis of navigation across species. Annu Rev Psychol.

[CR33] Fries P (2015). Rhythms for cognition: communication through coherence. Neuron.

[CR34] Gallistel CR (1980). The organization of action: a new synthesis.

[CR35] Gallistel CR (1981). Précis of Gallistel's The organization of action: a new synthesis. Behav Brain Sci.

[CR36] Gallup GG (1970). Chimpanzees: self-recognition. Science.

[CR37] Goodale MA, Ewert J-P, Capranica RR, Ingle DJ (1983). Visuomotor organization of pecking in the pigeon. Advances in vertebrate neuroethology.

[CR38] Graham P, Cheng K (2009). Ants use the panoramic skyline as a visual cue during navigation. Curr Biol.

[CR39] Graham P, Fauria K, Collett TS (2003). The influence of beacon-aiming on the routes of wood ants. J Exp Biol.

[CR40] Hamilton E, Pellicciotta N, Feriani L, Cicuta P (2019). Motile cilia hydrodynamics: entrainment versus synchronization when coupling through flow. Philos Trans of the R Soc B-Biol Sci.

[CR41] Hanson SJ, Timberlake W (1983). Regulation during challenge: a general model of learned performance under schedule constraint. Psychol Rev.

[CR42] Heinze S, Narendra A, Cheung A (2018). Principles of insect path integration. Curr Biol.

[CR43] Helfrich RF, Fiebelkorn IC, Szczepanski SM, Lin JJ, Parvizi J, Knight RT, Kastner S (2018). Neural mechanisms of sustained attention are rhythmic. Neuron.

[CR44] Hulse SH, Fowler H, Honig WK (1978). Cognitive processes in animal behavior.

[CR45] Iino Y, Yoshida K (2009). Parallel use of two behavioral mechanisms for chemotaxis in *Caenorhabditis elegans*. J Neurosci.

[CR46] Kelly DM, Spetch ML, Zentall TR, Wasserman EA (2012). Comparative spatial cognition: Encoding of geometric information from surfaces and landmark arrays. The Oxford handbook of comparative cognition.

[CR47] Kinosita H, Murakami A (1967). Control of ciliary motion. Physiol Rev.

[CR48] Koshland DE (1980). Bacterial chemotaxis in relation to neurobiology. Annu Rev Neurosci.

[CR49] Kramar M, Alim K (2021). Encoding memory in tube diameter hierarchy of living flow network. Proc Natl Acad Sci USA.

[CR50] Le Möel F, Wystrach A (2020). Opponent processes in visual memories: a model of attraction and repulsion in navigating insects’ mushroom bodies. PLoS Comput Biol.

[CR51] Legge ELG (2019). Comparative spatial memory and cue use: the contributions of Marcia L. Spetch to the study of small-scale spatial cognition. Behav Proc.

[CR52] Lent D, Graham P, Collett TS (2010). Image-matching during ant navigation occurs through saccade-like body turns controlled by learnt visual features. Proc Natl Acad Sci USA.

[CR53] Lent D, Graham P, Collett TS (2013). Visual scene perception in navigating wood ants. Curr Biol.

[CR54] Li B, Liu S, Hu D, Li G, Tang R, Song D, Lang Y, He J (2021). Electrocortical activity in freely walking rats varies with environmental conditions. Brain Res.

[CR55] Lisman JE, Jensen O (2013). The theta-gamma neural code. Neuron.

[CR56] Miller GA, Galanter E, Pribram KH (1960). Plans and the structure of behavior.

[CR57] Murray T, Kocsi Z, Dahmen H, Narendra A, Le Möel F, Wystrach A, Zeil J (2020). The role of attractive and repellent scene memories in ant homing (*Myrmecia croslandi*). J Exp Biol.

[CR58] Nakai T, Ando T, Goto T (2021). Biased reorientation in the chemotaxis of peritrichous bacteria *Salmonella enterica serovar* Typhimurium. Biophys J.

[CR59] Nolan SO, Zachry JE, Johnson AR, Brady LJ, Siciliano CA, Calipari ES (2020). Direct dopamine terminal regulation by local striatal microcircuitry. J Neurochem.

[CR60] Olton DS, Samuelson RJ (1976). Remembrance of places passed: Spatial memory in rats. J Exp Psychol Anim Behav Process.

[CR61] Pfeffer SE, Wahl VL, Wittlinger M, Wolf H (2019). High-speed locomotion in the Saharan silver ant, *Cataglyphis bombycina*. J Exp Biol.

[CR62] Pierce-Shimomura JT, Morse TM, Lockery SR (1999). The fundamental role of pirouettes in *Caenorhabditis elegans* chemotaxis. J Neurosci.

[CR63] Pomper U, Ansorge U (2021). Theta-rhythmic oscillation of working memory performance. Psychol Sci.

[CR64] Powers WT (1973). Behavior: the control of perception.

[CR65] Powers WT (1978). Quantitative analysis of purposive systems: some spadework at the foundations of scientific psychology. Psychol Rev.

[CR66] Ray SK, Valentini G, Shah P, Haque A, Reid CR, Weber GF, Garnier S (2019). Information transfer during food choice in the slime mold *Physarum polycephalum*. Front Ecol Evol.

[CR67] Reid CR, Latty T (2016). Collective behaviour and swarm intelligence in slime moulds. FEMS Microbiol Rev.

[CR68] Reid CR, Garnier S, Beekman M, Latty T (2015). Information integration and multiattribute decision making in non-neuronal organisms. Anim Behav.

[CR69] Roberts WA, Roitblat HL, Bever TG, Terrace HS (1984). Some issues in animal spatial memory. Animal cognition.

[CR70] Roberts C, Price GW, Middlemiss DN (2001). Ligands for the investigation of 5-HT autoreceptor function. Brain Res Bull.

[CR71] Roitblat HL, Terrace HS, Bever TG (1984). Animal cognition.

[CR72] Salmon M, Wyneken J (1987). Orientation and swimming behavior of hatchling loggerhead turtles *Caretta caretta* L. during their offshore migration. J Exp Mar Biol Ecol.

[CR73] Sanders H, Renno-Costa C, Idiart M, Lisman J (2015). Grid cells and place cells: an integrated view of their navigational and memory function. Trends Neurosci.

[CR74] Sanders H, Ji D, Sasaki T, Leutgeb JK, Wilson MA, Lisman JE (2019). Temporal coding and rate remapping: representation of nonspatial information in the hippocampus. Hippocampus.

[CR75] Savelli F, Knierim JJ (2019). Origin and role of path integration in the cognitive representations of the hippocampus: computational insights into open questions. J Exp Biol.

[CR76] Schultheiss P, Wystrach A, Schwarz S, Tack A, Delor J, Nooten SS, Bibost A-L, Freas CA, Cheng K (2016). Crucial role of ultraviolet light for desert ants in determining direction from the terrestrial panorama. Anim Behav.

[CR77] Shettleworth SJ (2010). Cognition, evolution, and behavior.

[CR78] Sleigh MA (1969). Coordination of the rhythm of beat in some ciliary systems. Int Rev Cytol.

[CR79] Smith-Ferguson J, Beekman M (2020). Who needs a brain? Slime moulds, behavioural ecology and minimal cognition. Adapt Behav.

[CR80] Solomon RL (1980). The opponent-process theory of acquired motivation: the costs of pleasure and the benefits of pain. Am Psychol.

[CR81] Spetch ML, Cheng K, MacDonald SE (1996). Learning the configuration of a landmark array: I. Touch-screen studies with pigeons and humans. J Comp Psychol.

[CR82] Spetch ML, Cheng K, MacDonald SE, Linkenhoker BA, Kelly DM, Doerkson SR (1997). Use of landmark configuration in pigeons and humans: II. Generality across search tasks. J Comp Psychol.

[CR83] Srivastava N, Clark DA, Samuel ADT (2009). Temporal analysis of stochastic turning behavior of swimming *C. elegans*. J Neurophysiol.

[CR84] Stanfield CL (2016) Principles of human physiology, Global edn. Pearson, Harlow

[CR85] Sterling P, Laughlin S (2015). Principles of neural design.

[CR86] Stone T, Webb B, Adden A, Weddig NB, Honkanen A, Templin R, Wcislo W, Scimeca L, Warrant E, Heinze S (2017). An anatomically constrained model for path integration in the bee brain. Curr Biol.

[CR87] Tanimoto Y, Kimura KD (2019). Neuronal, mathematical, and molecular bases of perceptual decision-making in *C. elegans*. Neurosci Res.

[CR88] Thermos K, Bagnoli P, Epelbaum J, Hoyer D (2006). The somatostatin sst(1) receptor: an autoreceptor for somatostatin in brain and retina?. Pharmacol Ther.

[CR89] Thota AK, Watson SC, Knapp E, Thompson B, Jung R (2005). Neuromechanical control of locomotion in the rat. J Neurotrauma.

[CR90] Timberlake W, Allison J (1974). Response deprivation: an empirical approach to instrumental performance. Psychol Rev.

[CR91] Tross J, Wolf H, Pfeffer SE (2021). Allometry in desert ant locomotion (*Cataglyphis albicans* and *Cataglyphis bicolor*)—does body size matter?. J Exp Biol.

[CR92] Van Houten J (1978). Two mechanisms of chemotaxis in *Paramecium*. J Comp Physiol.

[CR93] VanRullen R (2016). Perceptual cycles. Trends Cogn Sci.

[CR94] VanRullen R (2018). Attention cycles. Neuron.

[CR95] Venables PH (1960). Periodicity in reaction time. Br J Psychol.

[CR96] von Holst E, Mittelstaedt H (1950). Das Reafferenzprinzip. Naturwissenschaften.

[CR97] Wan KY, Goldstein RE (2016). Coordinated beating of algal flagella is mediated by basal coupling. Proc Natl Acad Sci USA.

[CR98] Wilson DM (1966). Insect walking. Annu Rev Entomol.

[CR99] Wystrach A (2021). Movements, embodiment and the emergence of decisions. Insights from insect navigation. Biochem Biophys Res Commun.

[CR100] Wystrach A, Schwarz S, Schultheiss P, Beugnon G, Cheng K (2011). Views, landmarks, and routes: how do desert ants negotiate an obstacle course?. J Comp Physiol A.

[CR101] Wystrach A, Lagogiannis K, Webb B (2016). Continuous lateral oscillations as a core mechanism for taxis in *Drosophila* larvae. Elife.

[CR102] Wystrach A, Schwarz S, Graham P, Cheng K (2019). Running paths to nowhere: repetition of routes shows how navigating ants modulate online the weights accorded to cues. Anim Cogn.

[CR103] Wystrach A, Buehlmann C, Schwarz S, Cheng K, Graham P (2020). Rapid aversive and memory trace learning during route navigation in desert ants. Curr Biol.

[CR104] Wystrach A, Le Moël F, Clement L, Schwarz S (2020b) A lateralised design for the interaction of visual memories and heading representations in navigating ants. HAL:hal-03052606 https://hal.archives-ouvertes.fr/hal-03052606

[CR105] Zeil J (2012). Visual homing: an insect perspective. Curr Opin Neurobiol.

